# Supplementary material for: Feeding Expressed Breast Milk Alters the Microbial Network of Breast Milk and Increases Breast Milk Microbiome Diversity over Time
Source: Microorganisms. 2024 Dec 25;13(1):12. doi: 10.3390/microorganisms13010012 (PMC11767962; doi:10.3390/microorganisms13010012)
Supplement: Supplementary file 1 [file microorganisms-13-00012-s001.zip › microorganisms-3357870-supplementary.pdf]

Feeding Expressed Breast Milk Alters the Microbial Network of Breast Milk and Increases Breast Milk Microbiome Diversity over Time

Supplementary Materials

Table S1: Enrollment criteria for the UPSIDE Cohort Study

Inclusion Criteria	Exclusion Criteria
>18 years of age	Plan to terminate pregnancy or give up child after birth
≤13 weeks gestation	Plan to use a non-approved study site for prenatal care/delivery
Prenatal care/delivery at medical facilities involved in study	Medical condition that would impede study procedures
Agreeable to study procedures after birth	Delivery prior to 37 weeks gestation
No history of psychiatric condition/recent history of drug use	Hormone medication use during pregnancy
English-speaking	Multiparous
	Drug use

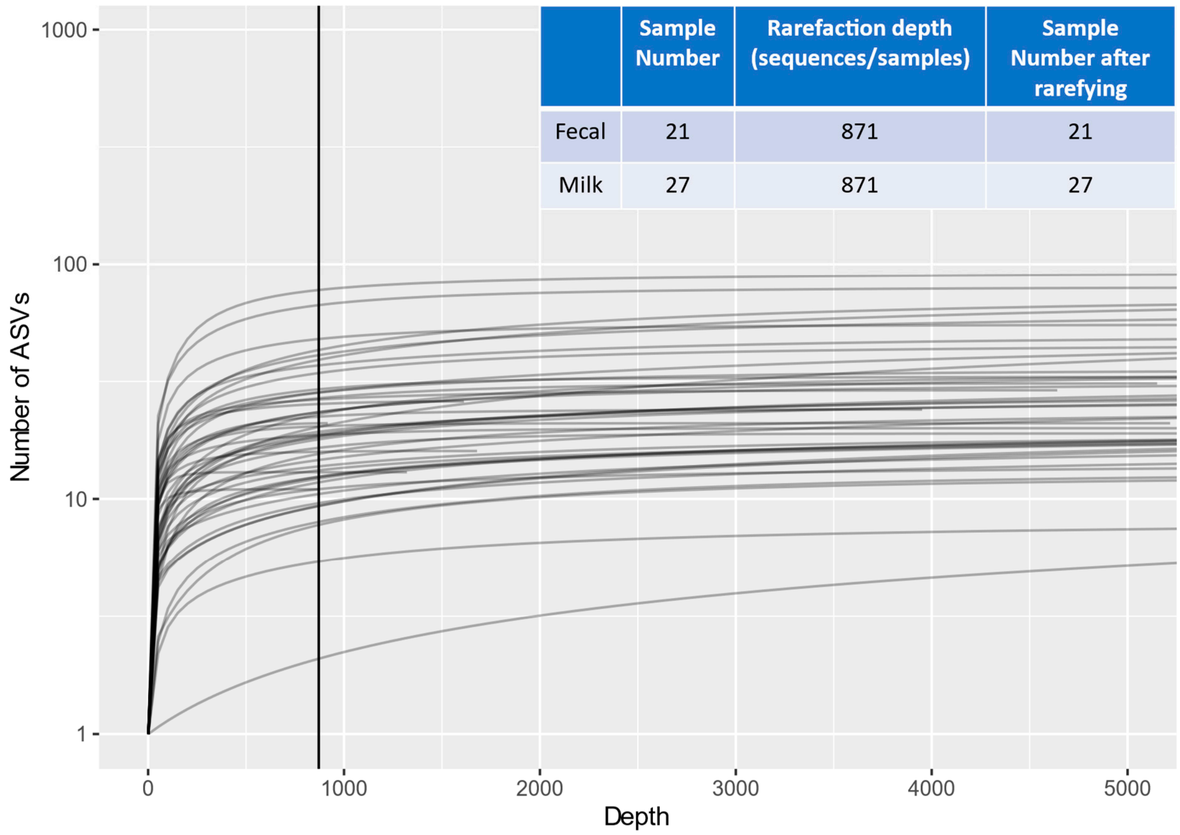

Figure S1: Rarefaction curves of bacterial microbiota in breast milk and infant feces. Rarifying at 871 ASVs per sample included all samples in the analyses.

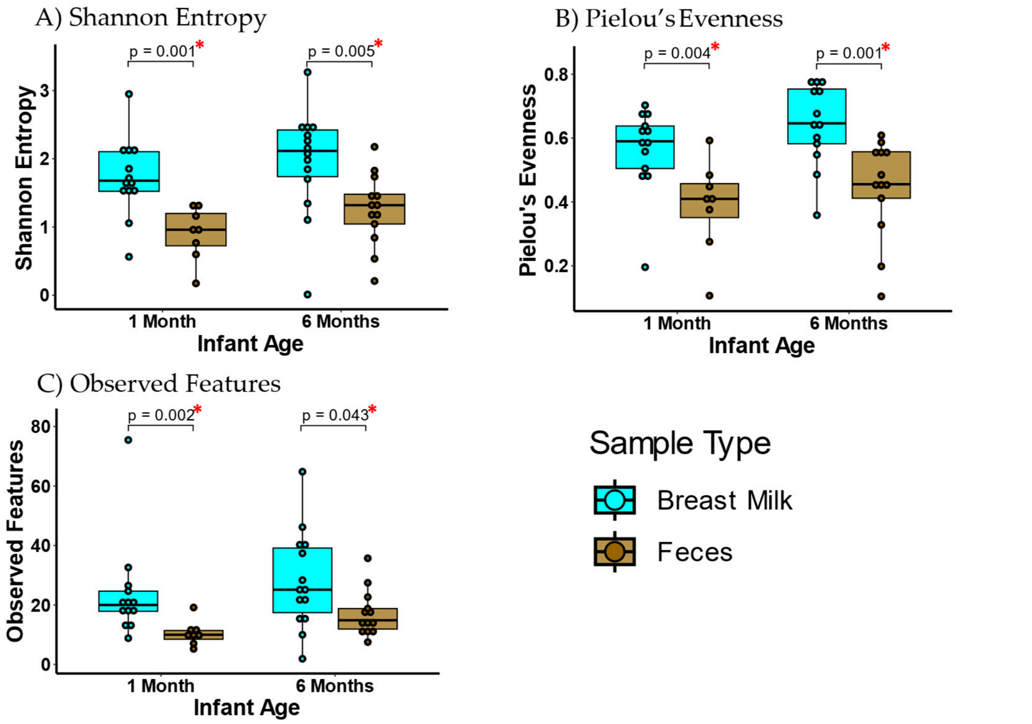

Figure S2: Bacterial microbiota alpha diversity between breast milk and infant feces in different age groups. A) Shannon Entropy, B) Pielou's Evenness, C) Observed Features. Each sample is represented as a point in the boxplot. Comparisons performed using Wilcoxon Rank Sum Test. Bacterial microbiota was more diverse in breast milk than in infant feces. * indicates significant *p* value of < 0.05

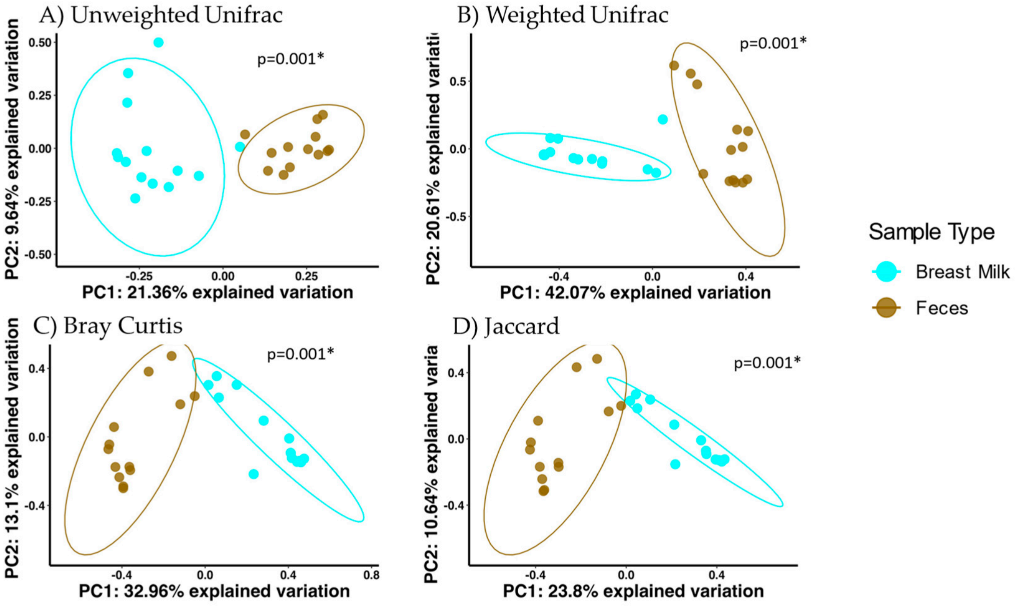

Figure S3: Bacterial microbiota beta diversity between breast milk and infant feces in 6-month-old infants. A) Unweighted Unifrac Distance, B) Weighted Unifrac Distance, C) Bray Curtis Distance, D) Jaccard Distance. Comparisons were performed by PERMANOVA test. Comparisons were performed by PERMANOVA test. Maternal breast milk microbiota differs from the infant's fecal microbiota. * indicates significant *p* value of < 0.05

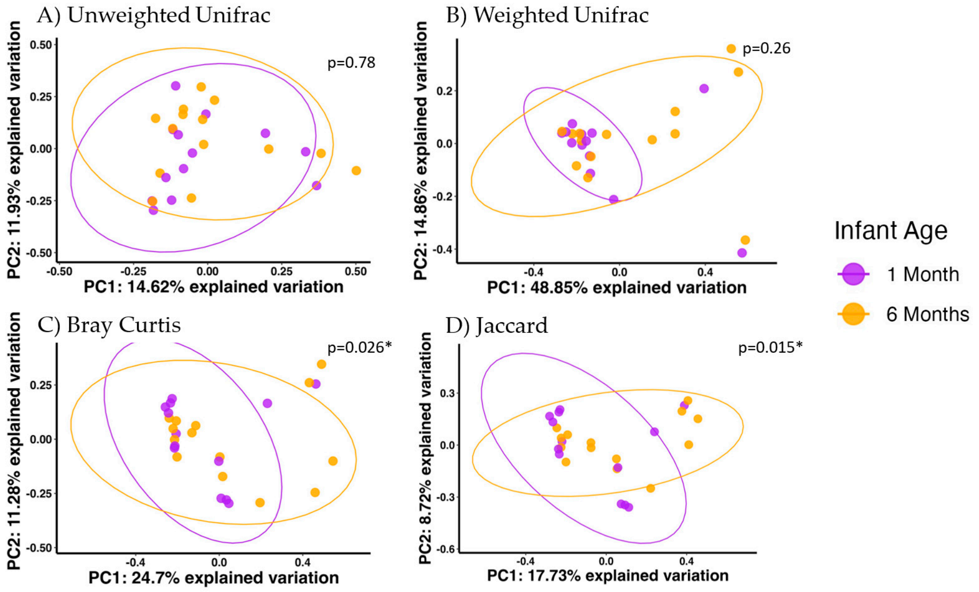

Figure S4: Bacterial microbiota beta diversity in breast milk between age groups. A) Unweighted Unifrac Distance, B) Weighted Unifrac Distance, C) Bray Curtis Distance, D) Jaccard Distance. All infants at 1 month were directly breastfed and providing expressed breast milk occurred between 1-6 months, only in the expressed breast milk group. Comparisons performed by PERMANOVA test. The structure of the breast milk bacterial microbiota differs significantly with age of the infant. * indicates significant *p* value of < 0.05

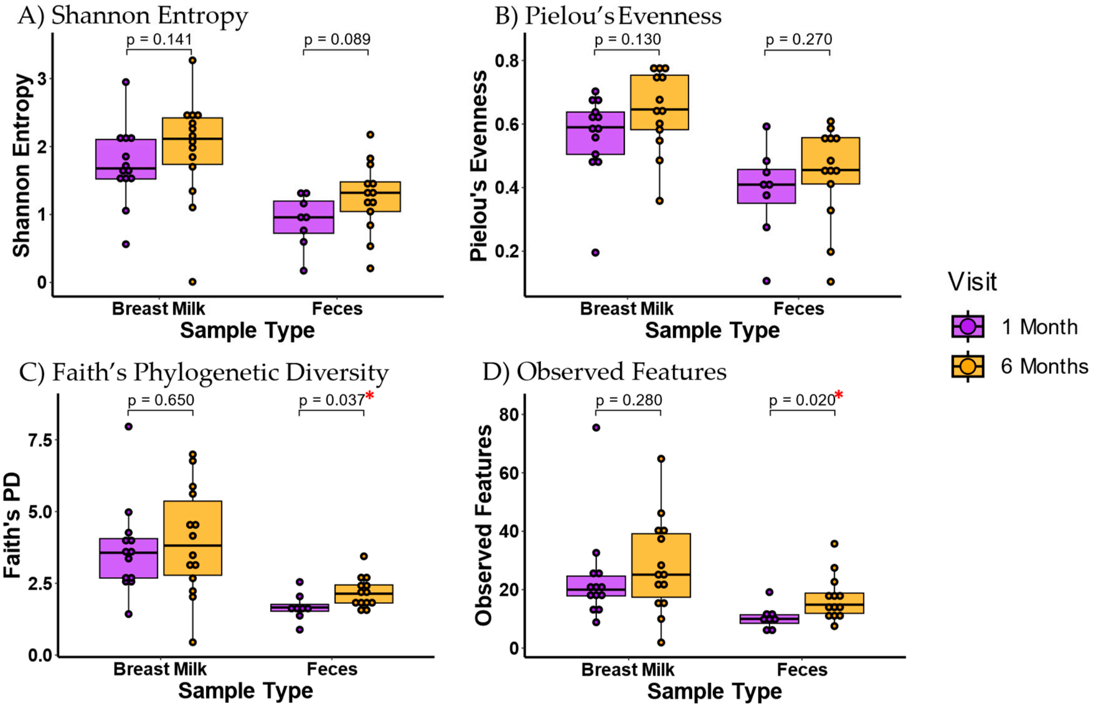

Figure S5: Bacterial microbiota alpha diversity in breast milk and feces between age groups. A) Shannon Entropy, B) Pielou's Evenness, C) Faith's Phylogenetic Diversity, D) Observed Features. All infants at 1 month were directly breastfed and providing expressed breast milk occurred between 1-6 months, only in the expressed breast milk group. Each sample is represented as a point in the boxplot. Comparisons performed using Wilcoxon Rank Sum Test. Infant fecal microbiota alpha diversity increased with age, but the breast milk microbiota alpha diversity did not. * indicates significant *p* value of < 0.05

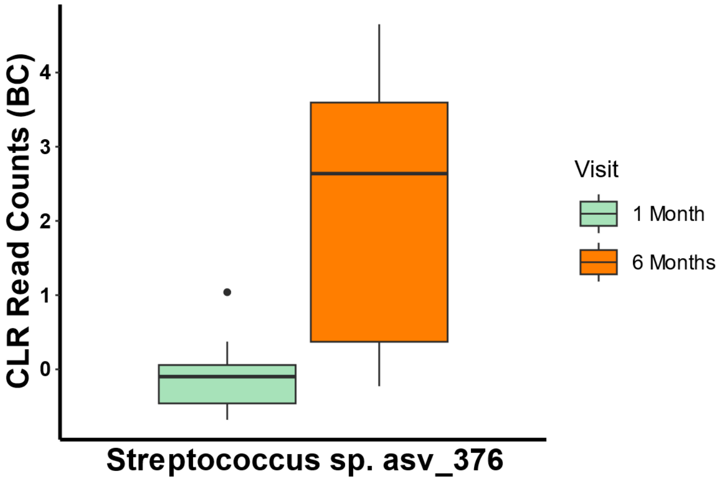

Figure S6: The significant discordant bacterial taxon in breast milk between infant age groups. Discordant bacteria determined using ANCOM. CLR= Central Log transformation Ratio; BC = Bias Corrected.

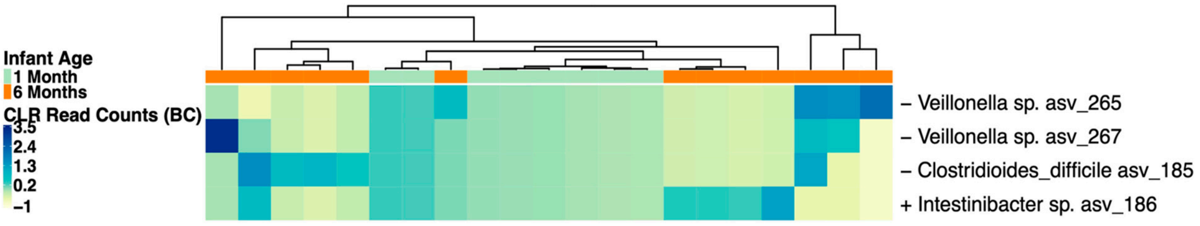

Figure S7: Unsupervised clustering of significant discordant bacterial taxa in feces between infants at 1 and 6 months of age. Discordant bacteria determined using ANCOM, and the figure shows log fold change: +: 1-2 Log fold change higher at 6 months than at 1 month; -: 1-2 Log fold change lower at 6 months;. CLR= Central Log transformation Ratio; BC = Bias Corrected.

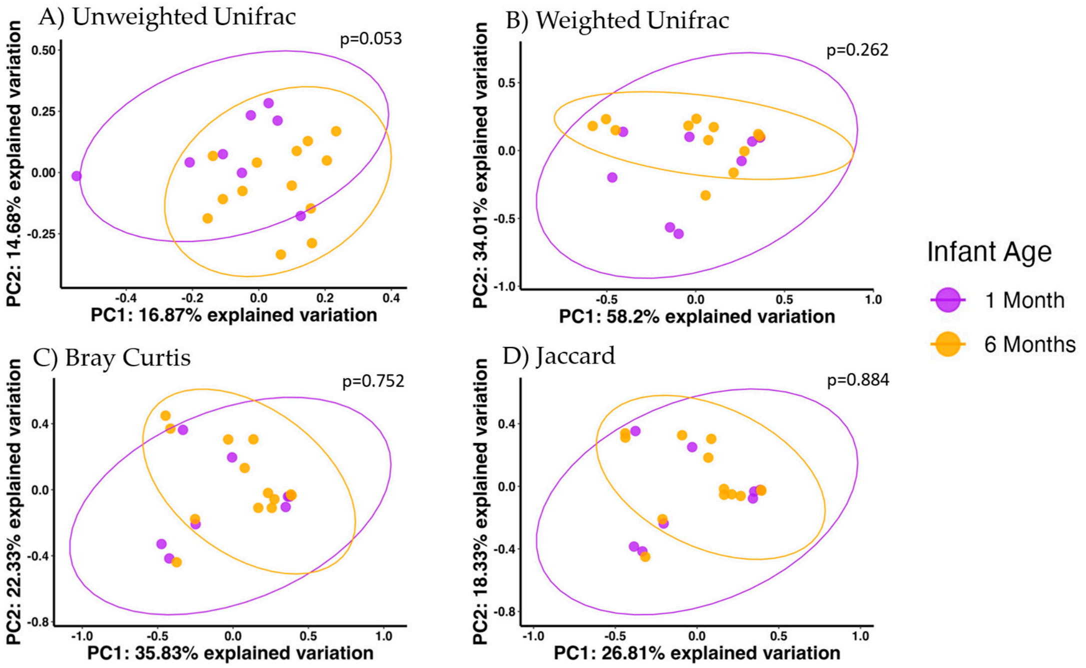

Figure S8: Bacterial microbiota beta diversity in infant feces between age groups. A) Unweighted Unifrac Distance, B) Weighted Unifrac Distance, C) Bray Curtis Distance, D) Jaccard Distance. All infants at 1 month were directly breastfed; feeding expressed breast milk occurred after 1 month of age. Comparisons performed by PERMANOVA test. Unweighted Unifrac comparing infant feces by age was trending towards significance, however no other beta diversity metrics were significantly different.

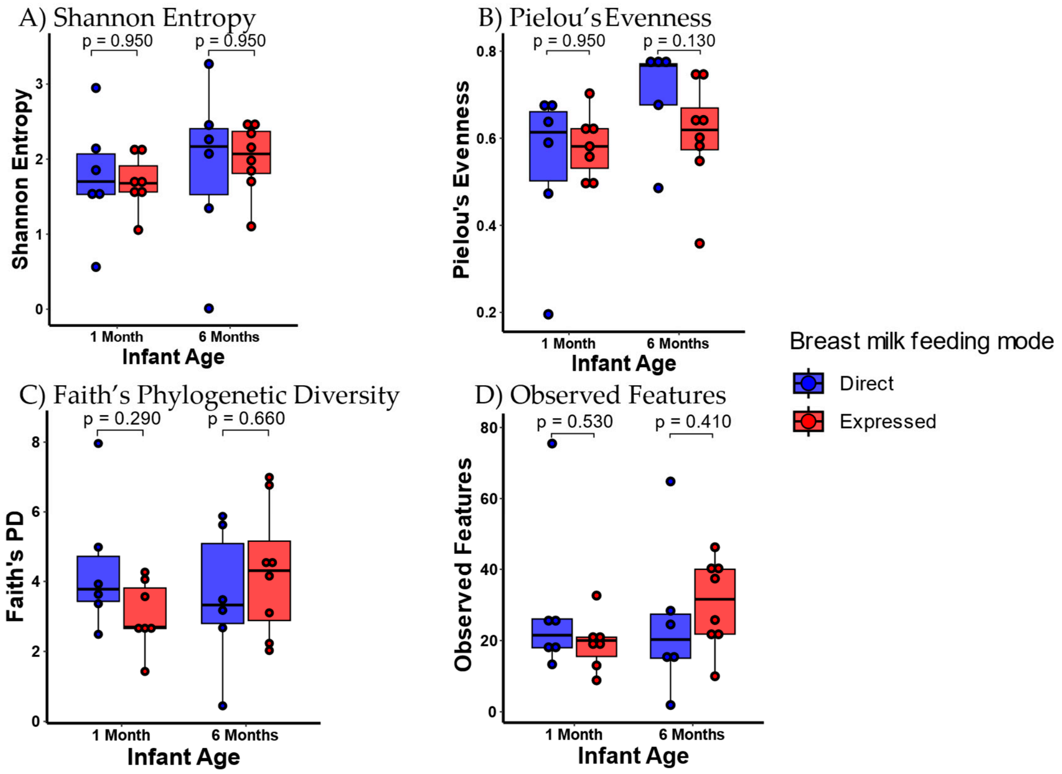

Figure S9: Bacterial alpha diversity in breast milk between breastfeeding methods at 1 and 6 months. A) Shannon Entropy, B) Pielou's Evenness, C) Faith's Phylogenetic Diversity, D) Observed Features. All infants at 1 month were directly breastfed and providing expressed breast milk occurred between 1-6 months, only in the expressed breast milk group. Each sample is represented as a point in the boxplot. Comparisons performed using Wilcoxon Rank-Sum Test. There were no significant differences in alpha diversity by breastfeeding method.

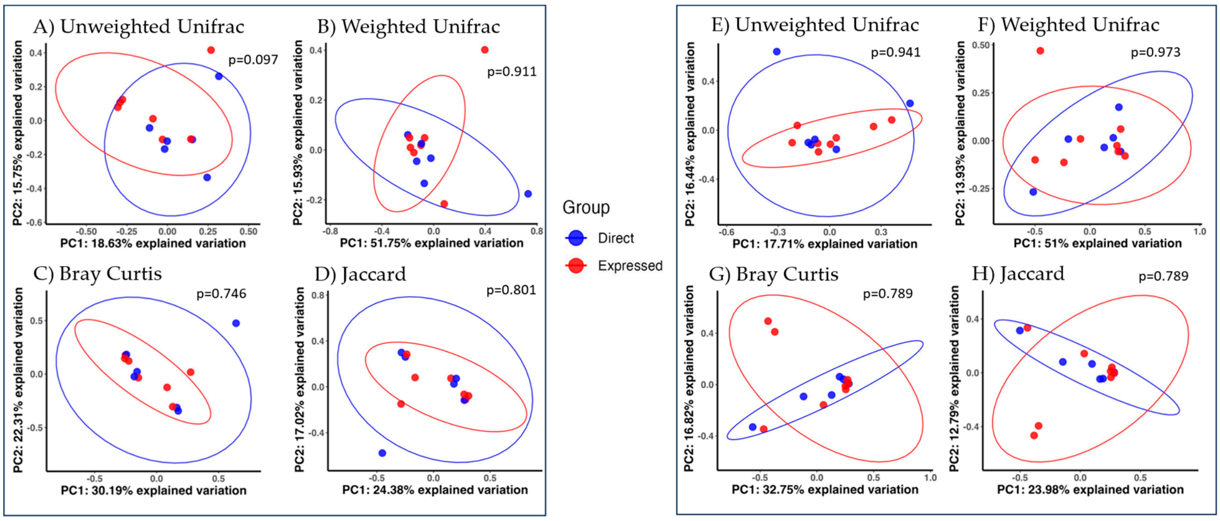

Figure S10: Bacterial beta diversity in breast milk between breastfeeding methods in different age groups. A) Unweighted Unifrac Distance- 1 month of age, B) Weighted Unifrac Distance- 1 month of age, C) Bray Curtis Distance- 1 month of age, D) Jaccard Distance- 1 month of age, E) Unweighted Unifrac Distance- 6 months of age, F) Weighted Unifrac Distance- 6 months of age, G) Bray Curtis Distance- 6 months of age, H) Jaccard Distance- 6 months of age. All infants at 1 month were directly breastfed and providing expressed breast milk occurred between 1-6 months, only in the expressed breast milk group. Comparisons performed by PERMANOVA test. No significant differences in the breast milk microbiota between breastfeeding methods at 1 month or 6 months of age.

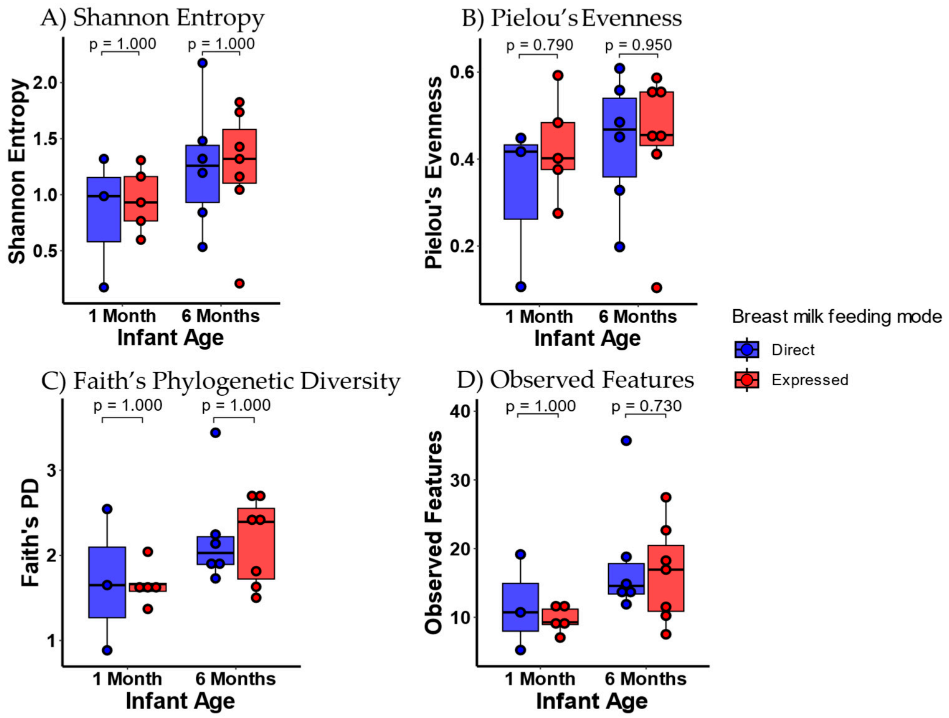

Figure S11: Bacterial alpha diversity in infant fecal microbiota between breastfeeding methods at 1 and 6 months. A) Shannon Entropy, B) Pielou's Evenness, C) Faith's Phylogenetic Diversity, D) Observed Features. All infants at 1 month were directly breastfed and providing expressed breast milk occurred between 1-6 months, only in the expressed breast milk group. Each sample is represented as a point in the boxplot. Comparisons performed using Wilcoxon Rank Sum Test. No significant difference in infant fecal alpha diversity noted between breastfeeding methods at either 1 or 6 months.

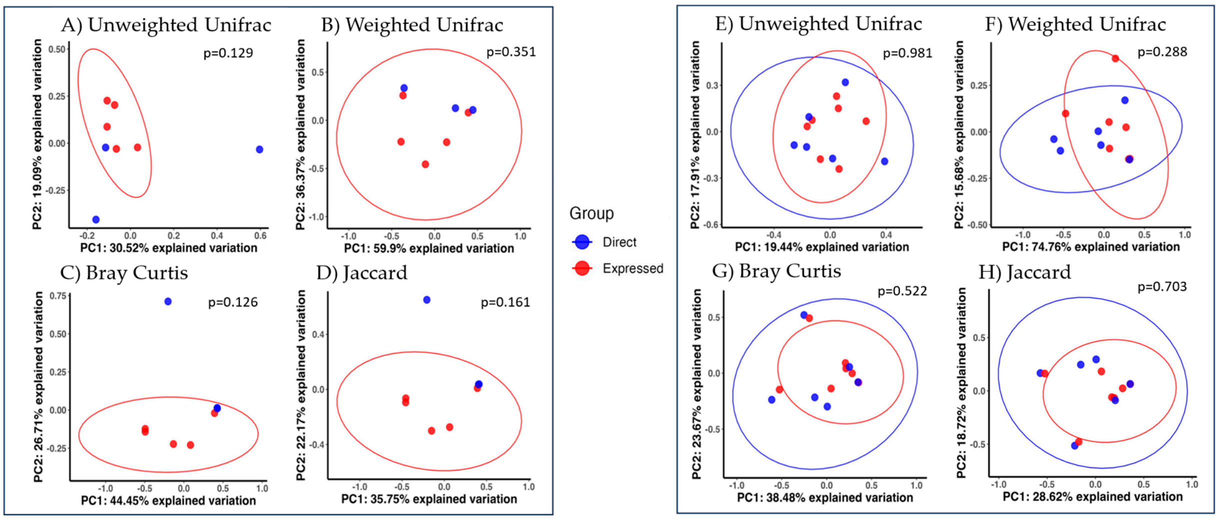

Figure S12: Bacterial beta diversity in infant feces between breastfeeding methods in different age groups. (A) Unweighted Unifrac Distance- 1 month of age, B) Weighted Unifrac Distance- 1 month of age, C) Bray Curtis Distance- 1 month of age, D) Jaccard Distance- 1 month of age, E) Unweighted Unifrac Distance- 6 months of age, F) Weighted Unifrac Distance- 6 months of age, G) Bray Curtis Distance- 6 months of age, H) Jaccard Distance- 6 months of age. All infants at 1 month were directly breastfed and providing expressed breast milk occurred between 1-6 months, only in the expressed breast milk group. Comparisons performed by PERMANOVA test. No significant differences at 1 or 6 months of age between breastfeeding methods.

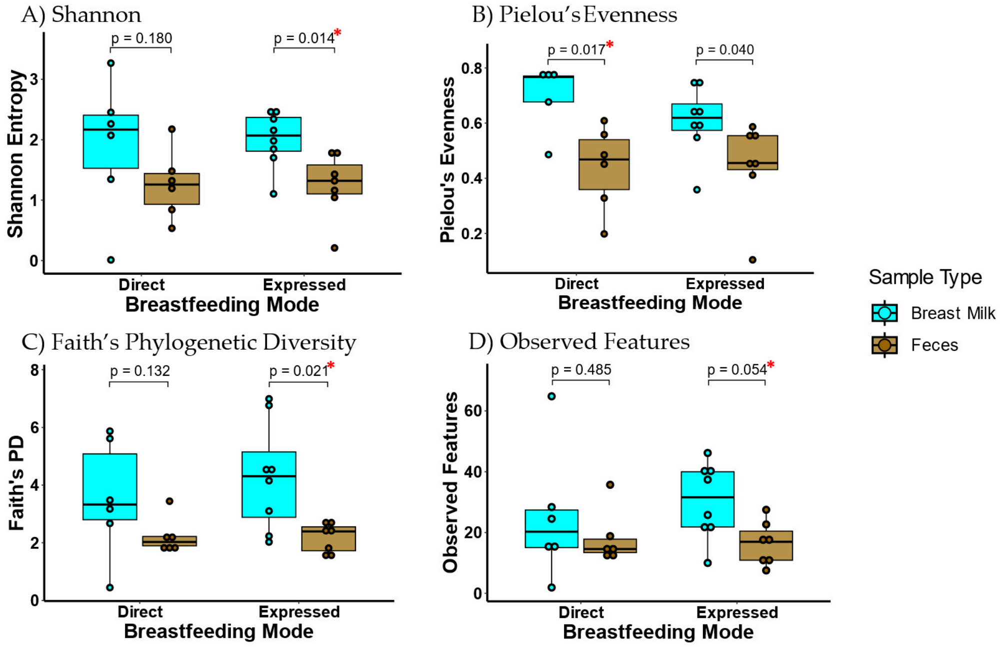

Figure S13: Bacterial microbiota alpha diversity between breast milk and infant feces in at 6 months of age, separated by breastfeeding mode. A) Shannon Entropy, B) Pielou's Evenness, C) Faith's Phylogenetic Diversity, D) Observed Features. Each sample is represented as a point in the boxplot. Comparisons performed using Wilcoxon Rank Sum Test. The differences in diversity between breast milk and infant feces are more marked in the expressed breast milk group. * indicates significant *p* value of < 0.05

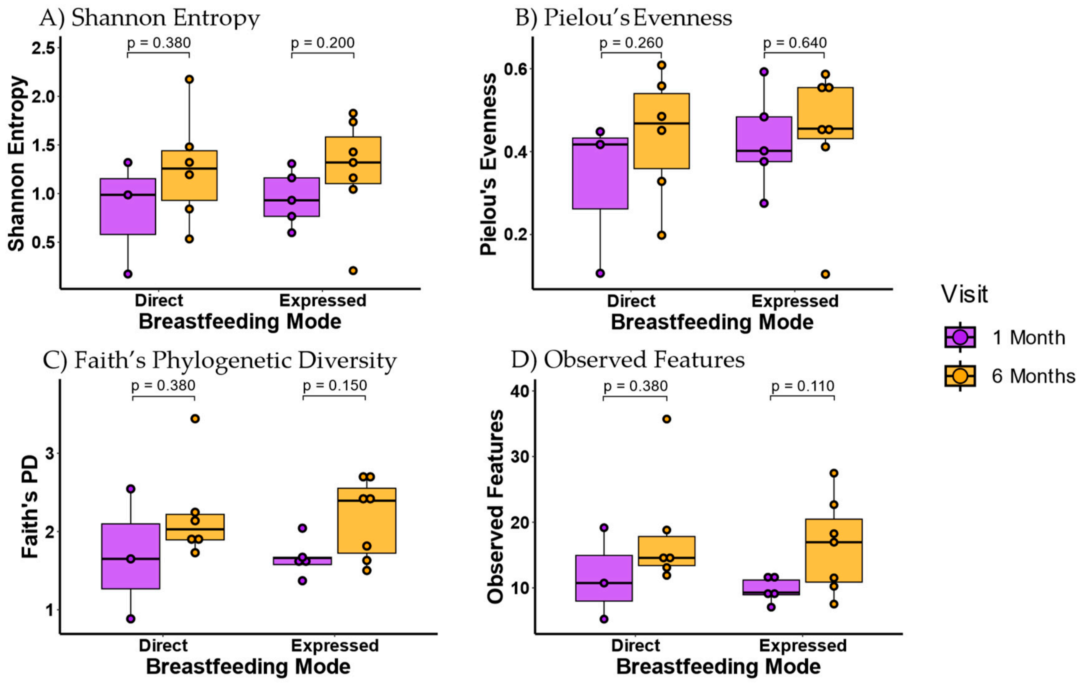

Figure S14: Bacterial microbiota alpha diversity in feces between age groups, separated by breastfeeding mode. A) Shannon Entropy, B) Pielou's Evenness, C) Faith's Phylogenetic Diversity, D) Observed Features. All infants at 1 month were directly breastfed and providing expressed breast milk occurred between 1-6 months, only in the expressed breast milk group. Comparisons performed by Wilcoxon Rank Sum Test. No significant differences with alpha diversity in feces between age groups.

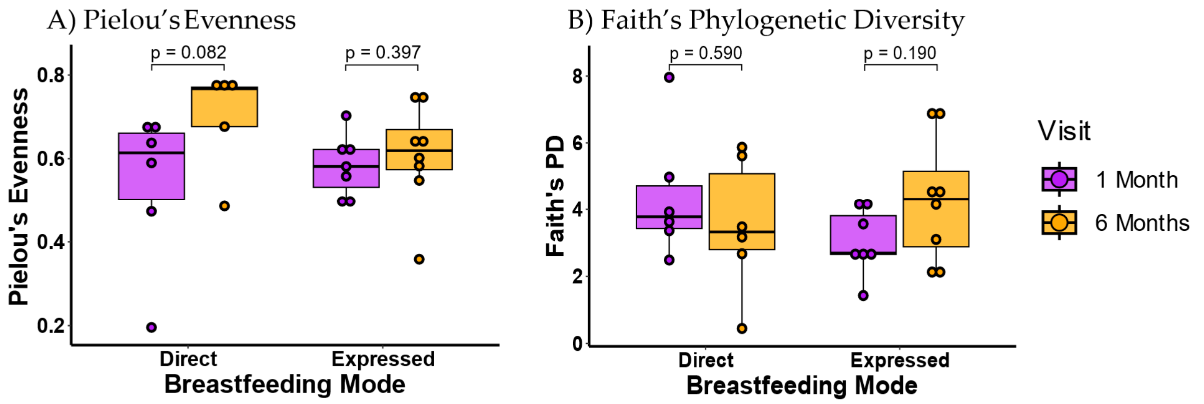

Figure S15: Bacterial microbiota alpha diversity in breast milk between age groups, separated by breastfeeding mode. A) Pielou's Evenness, B) Faith's Phylogenetic Diversity. All infants at 1 month were directly breastfed and providing expressed breast milk occurred between 1-6 months, only in the expressed breast milk group. Each sample is represented as a point in the boxplot. Comparisons performed using Wilcoxon Rank Sum Test. Breast milk microbiota alpha diversity increase with age only trending towards significance for direct group for Pielou's Evenness.
